# Supplementary material for: Towards a Sustainable Management of the Spotted-Wing Drosophila: Disclosing the Effects of Two Spider Venom Peptides on Drosophila suzukii
Source: Insects. 2023 Jun 7;14(6):533. doi: 10.3390/insects14060533 (PMC10299698; doi:10.3390/insects14060533)
Supplement: Supplementary file 1 [file insects-14-00533-s001.zip › insects-2398983-supplementary.pdf]

Supplementary Information

Towards a Sustainable Management of the Spotted-Wing Drosophila: Disclosing the Effects of Two Spider Venom Peptides on *Drosophila suzukii*

Laura Regalado [1,2], Sara Sario [1,2,*], Rafael J. Mendes [1,2], Javier Valle [3], Peta J. Harvey [4], Cátia Teixeira [2,†], Paula Gomes [2], David Andreu [3] and Conceição Santos [1,2]

1 iB2, Biology Department, Faculty of Sciences, University of Porto, 4169-007 Porto, Portugal; laura.regalado@fc.up.pt (L.R.); rafael.mendes@fc.up.pt (R.J.M.); csantos@fc.up.pt (C.S.)
2 LAQV-REQUIMTE, Faculty of Sciences, University of Porto, 4050-453 Porto, Portugal; ca.teixeira@gmail.com (C.T.); pgomes@fc.up.pt (P.G.)
3 Proteomics and Protein Chemistry Unit, Department of Medicine and Life Sciences, Pompeu Fabra University, 08002 Barcelona, Spain; javier.valle@upf.edu (J.V.); david.andreu@upf.edu (D.A.)
4 Institute for Molecular Bioscience, Australian Research Council Centre of Excellence for Innovations in Peptide and Protein Science, The University of Queensland, Brisbane, QLD 4072, Australia; peta.harvey@imb.uq.edu.au
* Correspondence: sara.sario@fc.up.pt
† Current address: Gyros Protein Technologies Inc., Tucson, AZ 85714, USA.

Material and methods

NMR spectroscopy

Nuclear magnetic resonance (NMR) analysis to determine the structural conformation of both SVPs was performed at the NMR Spectroscopy Facility at the Institute for Molecular Bioscience, University of Queensland, Brisbane, Australia. Each peptide (1 mg) was dissolved in 550 µL of 10% D_2O/90% H_2O at pH 3.2. Spectra were recorded at 298 K on a Bruker Avance III HD 600 MHz spectrometer equipped with a cryoprobe, including TOCSY (with an 80 s MLEV-17 spin lock), NOESY (mixing time of 200 ms) and natural abundance 15N HSQC (and also 13C HSQC for Hv1c). Solvent suppression was achieved using excitation sculpting. Spectra were processed using Topspin 3.6 and then analyzed using CcpNMR Analysis. Chemical shifts were referenced to internal 4,4-dimethyl-4-silapentane-1-sulfonic acid.

Results

Table S1. Analytical characterization of the final oxidized peptides. Purity and retention time (Rt) were obtained by HPLC, and m/z detected by LC-MS. In both analyses, elution was performed with a gradient of 5-50 % ACN (0.1% TFA) in water (0.1% TFA), 1 mL/min, for 15 min.

Peptide	Theorethical MW (Da)	Purity (%)	Rt (min)	m/z
Hv1c	3760.24	97.3	5.43	$[P+2H]^{2+}$ = 1881.15; $[P+3H]^{3+}$ = 1253.85; $[P+4H]^{4+}$ = 941.05; $[P+5H]^{5+}$ = 753.05
TRTX	3608.17	99	7.07	$[P+3H]^{3+}$ = 1203.35; $[P+4H]^{4+}$ = 903.00; $[P+5H]^{5+}$ = 722.60; $[P+6H]^{6+}$ = 602.35

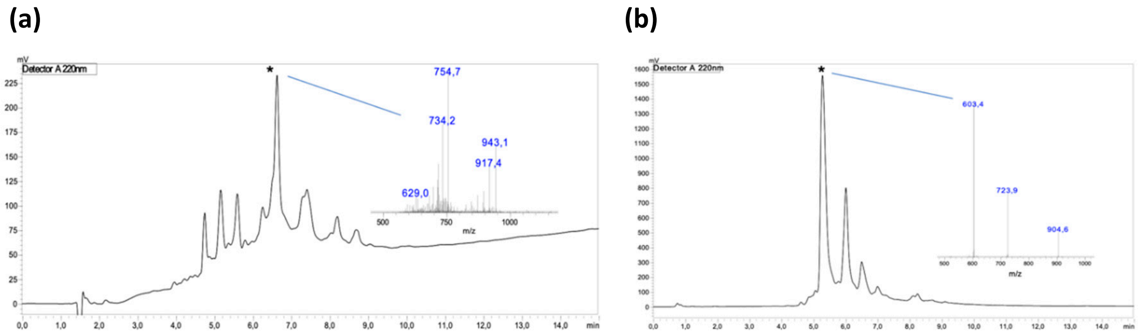

Figure S1. HPLC chromatogram and LC-MS spectra of crude products from solid-phase synthesis of peptides (**a**) Hv1c (Rt = 6.64 min) and (**b**) TRTX (Rt = 5.28 min). The symbol * highlights the major peak of each synthesis, which were confirmed by LC-MS analysis to be the desired peptides. The elution gradient used was 5-50% (for J-ACTX-Hv1c) and 15-50% (for µ-TRTX-Hhn2b) of solvent B into A (A: 0.1% TFA in water; B: 0.1% TFA in ACN), for 15 min, with a running flow of 1 mL/min and detection at λ = 220 nm.

(a)

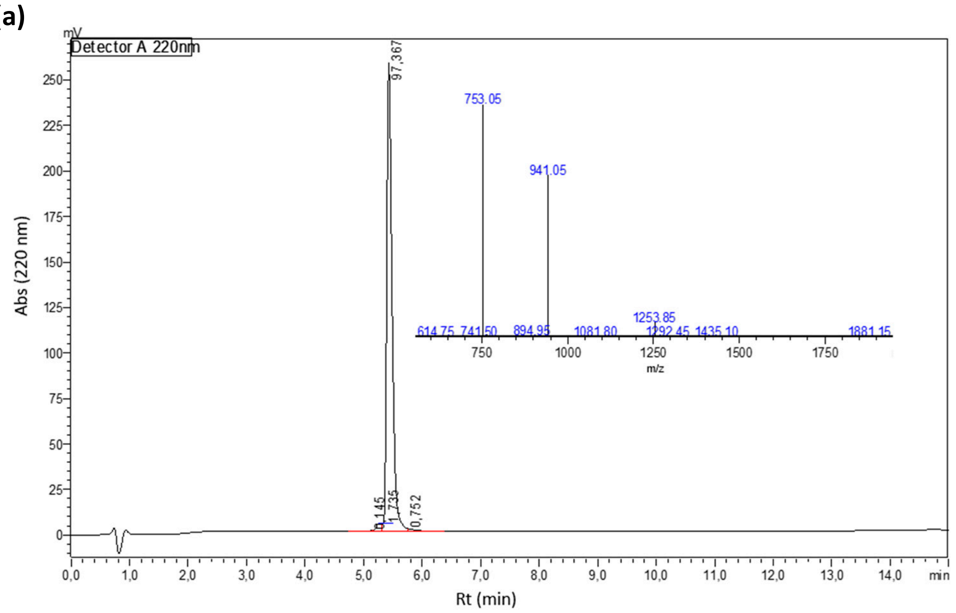

(b)

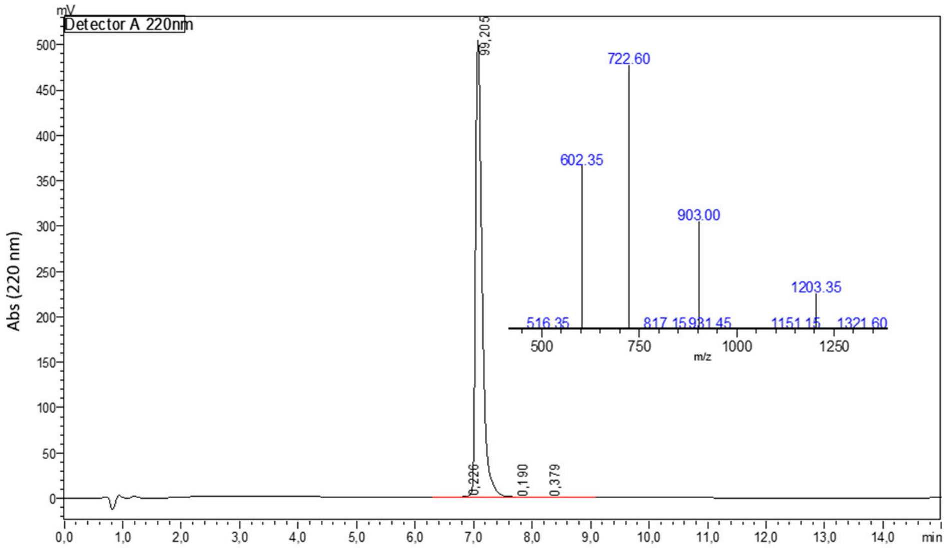

Figure S2. (**a**) chromatogram of purified Hv1c after oxidation (inserted is corresponding LC-MS spectrum): [P+2H]2+ = 1881.15; [P+3H]3+ = 1253.85; [P+4H]4+ = 941.05; [P+5H]5+ = 753.05); (**b**) chromatogram of purified TRTX after oxidation (inserted is the corresponding LC-MS spectrum: $[P+3H]^{3+}$ = 1203.35; $[P+4H]^{4+}$ = 903.00; $[P+5H]^{5+}$ = 722.60; $[P+6H]^{6+}$ = 602.35).

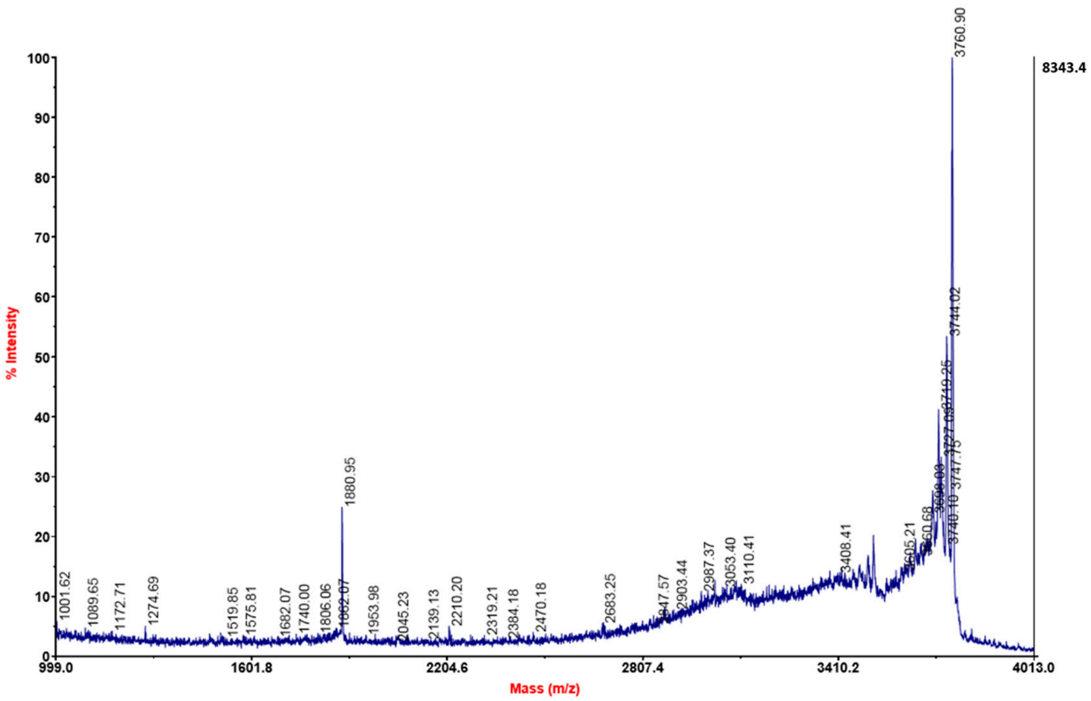

Figure S3. MALDI-TOF mass spectrum of oxidized Hv1c in positive ion mode. Mass spectrum was acquired from the mixture of equal volumes of the matrix solution (CHCA, 15 mg/mL in 50% MeCN in H_20) with peptide (1 mg/mL). The percentual intensities of the ions are shown on the y-axis and the ratio between mass and charge of the ions (m/z) are shown on the x-axis. m/z = 3760.90 corresponds to the peptide, and m/z = 1880.95 to peptide two times protonated (P+2H).

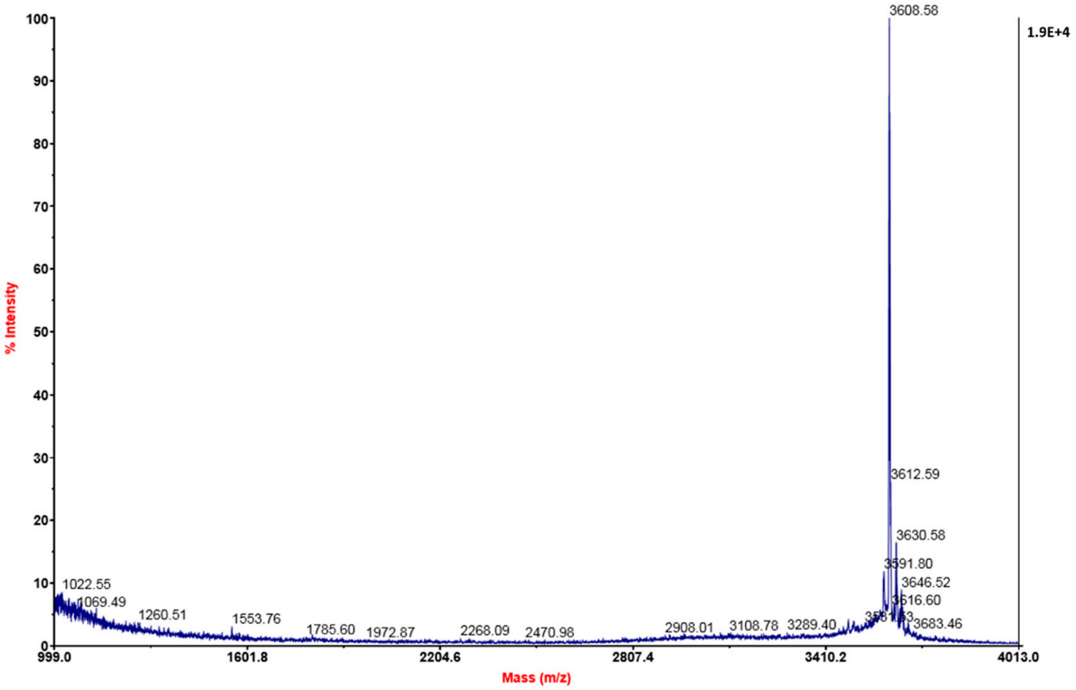

Figure S4. MALDI-TOF mass spectrum of oxidized TRTX in positive ion mode. Mass spectrum was acquired from the mixture of equal volumes of the matrix solution (CHCA, 15 mg/mL in 50% MeCN in H_20) with peptide (1 mg/mL). The percentual intensities of the ions are shown on the y-axis and the ratio between mass and charge of the ions (m/z) are shown on the x-axis. m/z = 3608.58 corresponds to the oxidized peptide.

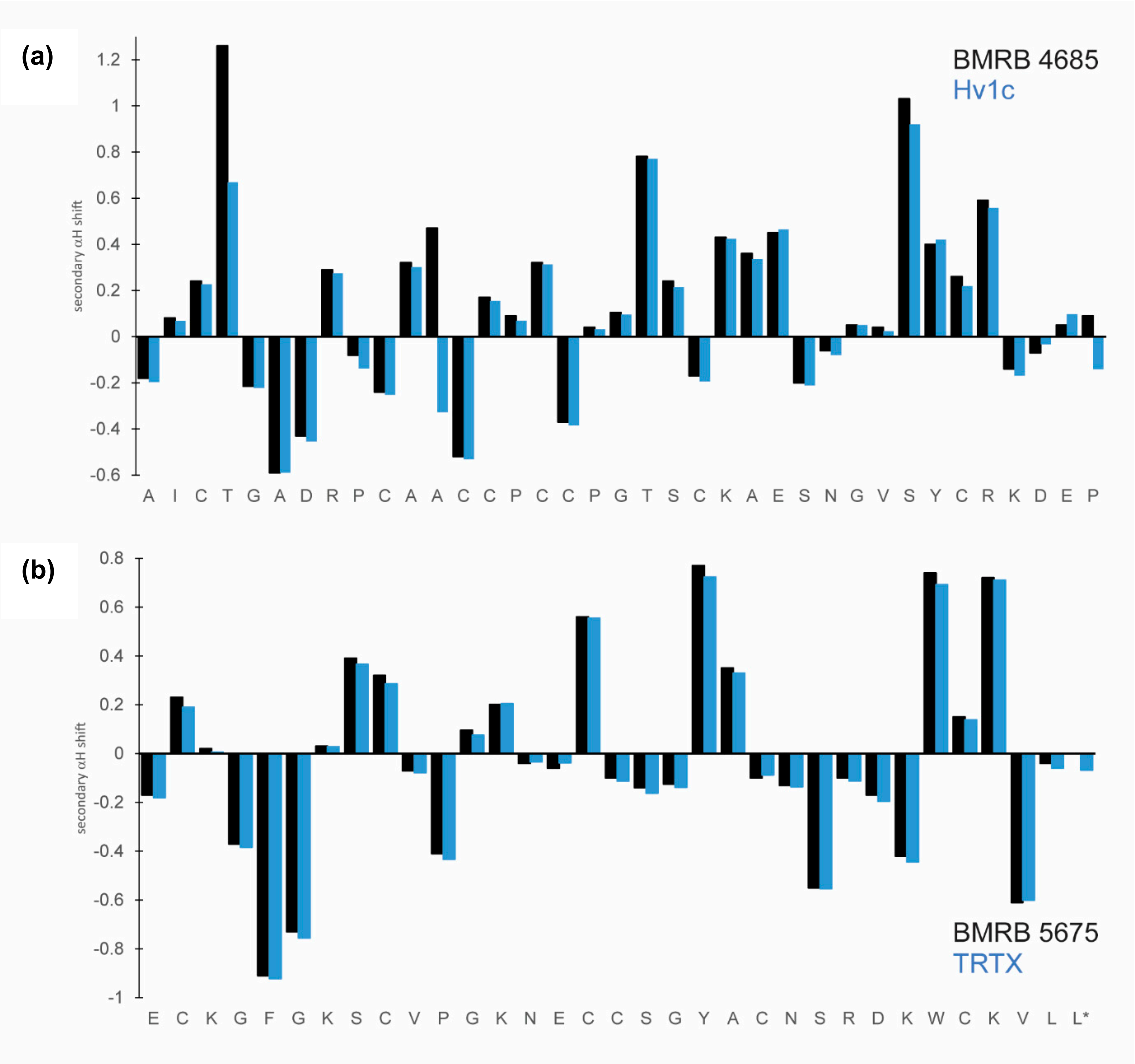

Figure S5. NMR analysis confirms correct folding and disulfide connectivities of synthetic peptides. Secondary alpha proton shift analysis, indicative of secondary structure elements, shows negligible differences compared to literature chemical shift data for both (**a**) Hv1c and (**b**) TRTX.

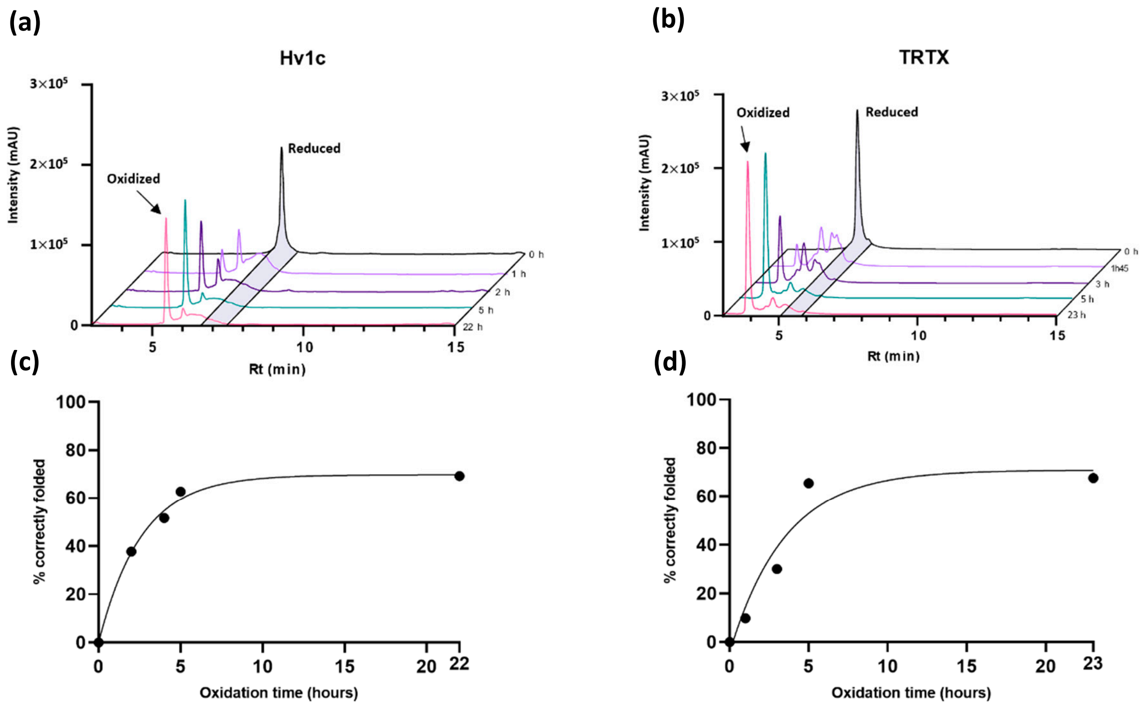

Figure S6. HPLC chromatograms of (**a**) Hv1c and (**b**) TRTX during oxidative folding. Peptides' oxidations were promoted at RT in an anaerobic environment, with 0.1 M NH_4OAc and 1 M Gd·HCl in the presence of GSH/GSSG, pH 7.8. The folding was monitored at multiple time points by HPLC and confirmed by LC-MS (data not shown). Arrows indicate the major peaks after oxidative folding, corresponding to the peptides' native conformation (later confirmed by NMR analysis); progress of (**c**) Hv1c and (**d**) TRTX folding reaction as monitored by integration of the major peak at each time point.

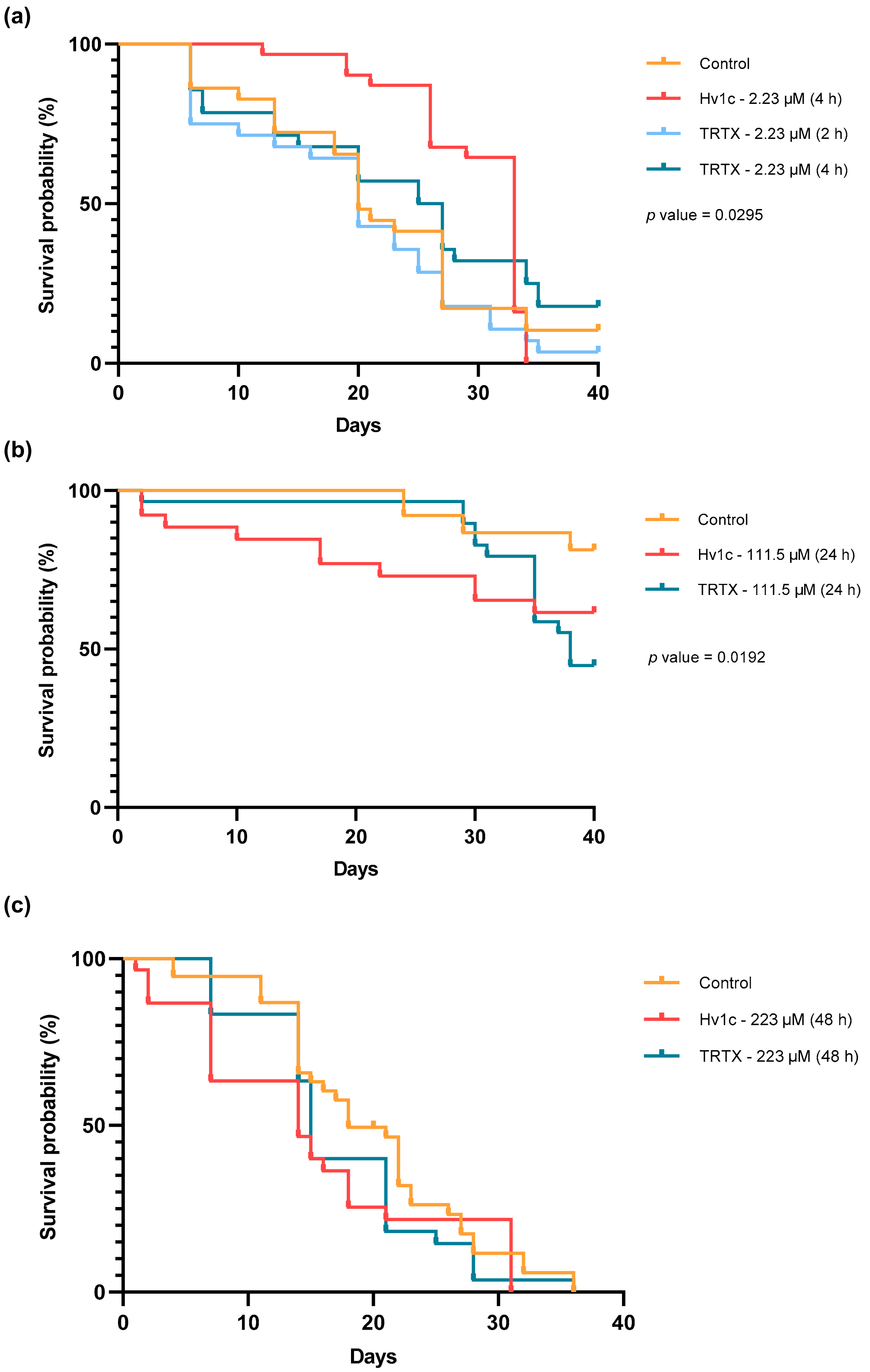

Figure S7. Kaplan-Meier survival curves of *D. suzukii* flies treated with (**a**) 2.23 μM of Hv1c (4 h) and TRTX (2 and 4 h), (**b**) SVPs at 111.5 μ M for 24 h, and (**c**) SVPs at 223 μM for 48 h. *Y*-axis indicates the probability of survival in percentage over time. Censoring is indicated in vertical marks. Only the results of the first 40 days after exposure to treatments are represented.
